# Molecular targeted therapy for metastatic colorectal cancer: current and evolving approaches

**DOI:** 10.3389/fphar.2023.1165666

**Published:** 2023-10-20

**Authors:** Furong Li, Yanping Lin, Rong Li, Xin Shen, Mengying Xiang, Guangrui Xiong, Ke Zhang, Tingrong Xia, Jiangyan Guo, Zhonghui Miao, Yedan Liao, Xuan Zhang, Lin Xie

**Affiliations:** ^1^ Department of Colorectal Surgery, The Third Affiliated Hospital of Kunming Medical University, Yunnan Cancer Hospital, Kunming, China; ^2^ Department of Gastroenterology and Internal Oncology, The Third Affiliated Hospital of Kunming Medical University, Yunnan Cancer Hospital, Kunming, China; ^3^ Department of Pathology, The Third Affiliated Hospital of Kunming Medical University, Yunnan Cancer Hospital, Kunming, China

**Keywords:** colorectal cancer, molecular targeted therapy, VEGF, epidermal growth factor receptor, RAS, Her-2

## Abstract

Colorectal cancer (CRC) represents 10% of all cancer types, making it the third leading cause of cancer-related deaths globally. Metastasis is the primary factor causing mortality in CRC patients. Approximately 22% of CRC-related deaths have metastasis present at diagnosis, with approximately 70% of these cases recurring. Recently, with the application of novel targeted drugs, targeted therapy has become the first-line option for individualized and comprehensive treatment of CRC. The management of these patients remains a significant medical challenge. The most prevalent targeted therapies for CRC in clinical practice focus on anti-vascular endothelial growth factor and its receptor, epidermal growth factor receptor (EGFR), and multi-target kinase inhibitors. In the wake of advancements in precision diagnosis and widespread adoption of second-generation sequencing (NGS) technology, rare targets such as BRAF V600E mutation, KRAS mutation, HER2 overexpression/amplification, and MSI-H/dMMR in metastatic colorectal cancer (mCRC) are increasingly being discovered. Simultaneously, new therapeutic drugs targeting these mutations are being actively investigated. This article reviews the progress in clinical research for developing targeted therapeutics for CRC, in light of advances in precision medicine and discovery of new molecular target drugs.

## 1 Introduction

Colorectal cancer (CRC) ranks as the third most common cancer in men and the second in women, with approximately 1.9 million new cases and 900,000 deaths worldwide in 2020 (Siegel et al., 2022). The annual incidence is almost 10% of all cancer cases, making it the second leading cause of cancer mortality (Siegel et al., 2020; Biller et al., 2021). Thus, CRC is a worldwide health issue regarding morbidity, death, use of healthcare, and rising costs for healthcare (Qiu et al., 2021; Kocarnik et al., 2022). Around 30% of CRC patients are found with distant metastases at diagnosis, contributing to up to 30% of deaths from cancer metastasis and recurrence (Beppu et al., 2014).

Treating CRC is a comprehensive treatment according to surgical resection, supplemented by chemotherapy, radiotherapy, targeted therapy, and others. The median survival time (Siegel et al., 2014) for patients with metastatic colorectal cancer (mCRC) has improved from 3.6–6 months to 24–28 months due to advancing chemotherapies, molecularly targeted treatments, immune checkpoint inhibitors, and evolving surgical techniques for treating liver and lung metastatic lesions. The advancements in chemotherapy have hit a ceiling of effectiveness, and the pivotal role in enhancing outcomes for advanced CRC has now shifted toward molecular targeted therapy. This method serves as the premier choice for a personalized, comprehensive treatment approach to CRC, offering patients a marked improvement in survival benefits. We study the progress of clinical research on developing targeted therapeutics for mCRC patients in the era of precision medicine and of medication research for new molecular targets, which provides evidence-based medicine for individualized precision treatment and precision targeted therapy and new therapeutic tools for mCRC patients with several recommendations of treatment, with the ultimate aim of improving their survival and bettering their quality of life.

Targeted therapies for CRC mainly include anti-vascular endothelial growth factor/vascular endothelial growth factor receptor (VEGF/VEGFR), epidermal growth factor receptor (EGFR), and multi-target kinase inhibitors, as well as their rare and uncommon targets (Table 1).

**TABLE 1:** Key phase 2 and 3 trials of molecular targeted therapy of mCRC patients.

Drugs	Experiment	Phase	Number of patients	Treatment regimen	Primary endpoints	Number of approved treatment lines
Anti-angiogenesis						
Bevacizumab	Hurwitz^[13]^	Phase 3	vs. 411	IFL plus bevacizumab vs. IFL plus placebo	PFS: 10.6 vs. 6.2 m	First-line
					OS: 20.3 vs. 15.6 m	
Aflibercept	VELOUR^[16]^	Phase 3	613 vs. 613	Aflibercept + FOLFIRI vs. FOLFIRI	PFS: 6.90 vs. 4.67 m	Second-line
					OS: 13.5 vs. 12.06 m	
Ramucirumab	RAISE^[15]^	Phase 3	536 vs. 536	Ramucirumab + FOLFIRI vs. FOLFIRI	PFS: 5.7 vs. 4.5 m	Second-line
					OS: 13.3 vs. 11.7 m	
Regorafenib	CORRECT^[17]^	Phase 3	505 vs. 255	Regorafenib vs. placebo	PFS: 1.9 vs. 1.7 m	Three-line
					OS: 6.4 vs. 5.0 m	
Fruquintinib	FRESCO-2^[18]^	Phase 3	458 vs. 229	Fruquintinib vs. placebo	PFS: 3.7 vs. 1.8 m	Three-line
					OS: 7.4 vs. 4.8 m	
Anti-EGFR monoclonal antibody						
Cetuximab	CRYSTAL^[21]^	Phase 3	367	FOLFIRI/cetuximab vs. FOLFIRI	ORR: 66.3 vs. 38.6 m	First-line
					PFS: 11.4 vs. 8.4 m	
					OS: 28.4 vs. 20.2 m	
Panitumumab	PRIME^[23]^	Phase 3	656 vs. 512	FOLFOX/panitumumab vs. FOLFOX	ORR: 55% vs. 48%	First-line
					PFS: 10.1 vs. 7.9 m	
					OS: 25.8 vs. 20.2 m	
BRAF inhibitors						
Vemurafenib	NCT00405587^[31]^	Phase 2	21	Vemurafenib	ORR: 5.0%	Second-line
					PFS: 2.1 m	
					OS: 7.7 m	
Dabrafenib	NCT01750918^[32]^	Phase 2	20 vs. 91 vs. 31	Dabrafenib plus Panitu_x005fmumab (D + P) ± trametinib (D + P + T)/(P + T):D + P vs. D + P + T vs. P + T	ORR: 10.0% vs. 21.0% vs. 0.0%	Second-line
					PFS: 3.5 vs. 4.2 vs. 2.6 m	
					OS: 13.2 vs. 9.2 vs. 9.1 m	
Encorafenib	BEACON^[33]^	Phase 3	224 (triplet);	Encorafenib plus Cetuxi_x005fmab ± binimetinib (triplet and doublet) vs. irinotecan plus cetuximab (control)	ORR: 26.8% and 19.5% vs. 1.8%	Second-line
			220 (doublet);		PFS: 4.5 and 4.3 vs. 1.5 m	
			221 (control)		OS: 9.3 and 9.3 vs. 5.9 m	
Anti-HER-2						
Trastuzumab	HERACLES^[37]^	Phase 2	27	Trastuzumab and lapatinib	PFS: 21 w	Second-line
					OS: 46 w	
Pertuzumab	MyPathway^[38]^	Phase 2	57	Pertuzumab and trastuzumab	DCR: 32%	Second-line
					PFS: 5.9 m	
Lapatinib	HERACLES^[37]^	Phase 2	27	Trastuzumab and lapatinib	PFS: 21 w	Second-line
					OS: 46 w	
Tucatinib	MOUNTAINEER^[41]^	Phase 2	117	Trastuzumab	PFS: 8.2 m	Second-line
					OS: 24.1 m	
KRAS G12C inhibitor						
Sotorasib	CodeBreaK100^[51]^	Phase 1	129, of which 42 had KRAS G12C mCRC	Sotorasib	ORR: 78.3%	Second-line
					PFS: 7.1 m	
					OS: 4.0 m	
Adagrasib	KRYSTAL-1^[52]^	Phase 2	45	Adagrasib monotherapy (phase 1/2)	ORR: 87.0% vs. 100.0%	Second-line
			28	Adagrasib plus cetuximab (first data cut-off analysis)	PFS: 22.0 vs. 43 m	
NTRK gene fusion						
Entrectinib^[58]^	—	Phase 2	10	Entrectinib	ORR: 20%	Second-line
Larotrectinib^[59]^	—	Phase 2	19	Larotrectinib	ORR: 47%	Second-line
					DCR: 89%	
RET inhibitors						
Selpercatinib	LIBRETTO-001^[64]^	Phase 2	10	Selpercatinib	ORR: 20.0%	Second-line
					PFS: 13.2 m	
					DOR: 24.5 m	

ORR, objective response rate; PFS, progression-free survival; OS, overall survival; DFS, disease-free survival; DCR, disease control rate; DOR, duration of overall response.

## 2 Anti-VEGF/VEGFR and kinase inhibitors: bevacizumab, ramolutumab, aflibercept, regorafenib, and fruquintinib

Angiogenesis is a characteristic of the onset and progression of all solid tumors, such as CRC (Folkman, 2007). Vascular regeneration plays a crucial part in the pathophysiology of tumors. The maximum diameter of tumors without the nutrients provided by the vascular system does not exceed 2 mm. The vascular network of tumors is formed by tumor regeneration, and this process is turned on by stimulating angiogenic factors. The VEGF pathway has been widely studied as a very important factor in the vascular regeneration phase, which includes the VEGF-A, VEGF-B, VEGF-C, VEGF-D, and placental growth factor (PLGF) (Ferrara et al., 2003; Cao, 2009). Three VEGF receptors (VEGFRs), namely, VEGFR1, VEGFR2, and VEGFR3, are high-affinity membrane tyrosine kinase receptors that transmit biological signals. VEGFR1 and VEGFR2 are mainly expressed in vascular endothelial cells, while VEGFR3 is primarily expressed in lymphatic vascular endothelial cells. The binding of a VEGF with the extracellular region of a receptor causes a change in its molecular conformation due to receptor dimerization, which results in autophosphorylation of receptor tyrosine residues that stimulates various sequences of signaling pathways that have roles in endothelial cell budding, migration, vascular permeability, and tumor cell survival (Joukov et al., 1997; Cao et al., 1998). The binding of the VEGF to VEGFR leads to receptor dimerization and autophosphorylation, subsequently activating downstream signaling pathways. These pathways, which include the PI3K/Akt and Ras/Raf/MEK/ERK pathways, stimulate angiogenesis and the permeability of blood vessels. Monoclonal antibodies such as bevacizumab, ramolutumab, and aflibercept have been authorized for treating mCRC. Bevacizumab is a recombinant human immunoglobulin G monoclonal antibody with a high affinity for selectively binding to human VEGF-A, which prevents the binding of a VEGF to its receptor and counteracts VEGF biology. It is the first anti-angiogenic drug authorized for treating mCRC. Multiple phase 2 and phase 3 research have revealed that the addition of bevacizumab to 5-Fu-based chemotherapy protocols significantly improves progression-free survival (PFS) and overall survival (OS) in mCRC patients (Saltz et al., 2008). Bevacizumab is appropriate for both first- or second-line treatments, and depending on the hyperprogressive strategy, it can be used across lines of therapy by switching chemotherapy to both first- and second-line therapies. Two other monoclonal antibodies (ramolutumab and aflibercept) are used in second-line therapy combined with FOLFIRI, after the progression of bevacizumab combined with FOLFOX therapy. Ramolutumab is a humanized monoclonal antibody that selectively binds to the extracellular region of VEGFR2 and blocks VEGFR2 phosphorylation; it is the only monoclonal antibody to VEGFR2 that has been marketed worldwide (Fuchs et al., 2014; Wilke et al., 2014). Aflibercept, a fusion protein formed by the recombination of extracellular region binding domains of human VEGF receptors 1 and 2 with the Fc segment of human immunoglobulin, is a new anti-VEGF drug that further inhibits neovascularization by binding tightly to VEGF and reducing vascular permeability (Van Cutsem et al., 2012).

Small molecule tyrosine kinase inhibitors authorized for treating mCRC include regorafenib and fruquintinib, which are effective enough as a third-line therapy once disease has progressed. Regorafenib (Grothey et al., 2013), an oral multikinase inhibitor, inhibits angiogenesis (VEGFR 1–3 and Tie2), tumorigenesis (KIT, RET, RAF1, and BRAF), and the tumor microenvironment (platelet-derived and fibroblast growth factor receptors), which leads to suppression inhibition of tumorigenesis, tumor neovascularization, and maintenance of tumor microenvironment signaling. Fruquintinib (Dasari et al., 2021) is a highly selective anti-tumor angiogenesis inhibitor developed by China, the main target of which is VEGFR kinase family VEGFR1/2/3. Fruquintinib inhibits VEGFR kinase activity at the molecular level and VEGFR2/3 phosphorylation at the cellular level, endothelial cell proliferation and lumen formation, tumor angiogenesis, and tumor growth. Unfortunately, even after two decades of intense translational and clinical research, no biomarkers to predict the activity or effectiveness of anticancer antiangiogenic medications have been established. Consequently, when compared with chemotherapy, these medicines continue to be administered to unselected mCRC patients.

## 3 Anti-EGFR monoclonal antibodies: panitumumab and cetuximab

Numerous specific ligands, which include the epidermal growth factor (EGF), transforming growth factor alpha (TGF-α), bidirectional regulatory proteins, and epi-regulatory proteins, can trigger the activation of the epidermal growth factor receptor (EGFR). The binding of these ligands to the extracellular structural domain of EGFR induces alterations in the receptor conformation, leading to the phosphorylation of distinct tyrosine residues within the EGFR’s intracellular structural domain. Consequently, this activates an intricate signaling cascade encompassing the RAS-RAF-MEK-MAPK and PTEN-PI3K-AKT mTOR pathways, which crucially govern the aggression of cancer cells and stimulate angiogenesis (Normanno et al., 2009).

The EGFR is expressed in healthy and cancer epithelial cells and plays a crucial part in the biological process of tumor development. It is overexpressed in 40%–70% of CRC cells and is significantly related to elevated metastatic probability and reduced survival in CRC. Currently, the Food and Drug Administration (FDA) has authorized the use of EGFR inhibitors cetuximab and panitumumab. Cetuximab is a human murine immunoglobulin antibody IgG antibody with a strong affinity for the extracellular binding domain of the EGFR that suppresses the binding of endogenous ligands competitively. Cetuximab promotes antibody-dependent cell-mediated cytotoxicity, activates pro-apoptotic molecules, and possesses a powerful synergistic impact when combined with radiotherapy or chemotherapy. In the previously published CRYSTAL study (Van Cutsem et al., 2009), the first-line treatment with cetuximab for CRC expressing wild-type RAS showed outstanding results, with a significant increase in PFS and OS, along with a 15% decrease in the risk of disease progression and mortality.

Panitumumab is a fully humanized IgG2 monoclonal antibody with a strong potential for the extracellular binding domain of the EGFR that competes with endogenous ligands to prevent ligand-induced autophosphorylation of the EGFR carboxyl residues and associated downstream signaling (Amado et al., 2008). Panitumumab exerts its anticancer actions primarily by promoting apoptosis and reducing proliferation, invasiveness, angiogenesis, and metastasis. In 2006, the FDA authorized Vectibix (panitumumab) as monotherapy for treating mCRC expressing EGFR for which fluoropyrimidine, oxaliplatin, and irinotecan chemotherapy had been previously ineffective. In 2014, the FDA expanded the indication and approved Vectibix plus FOLFOX for first-recommended therapy of mCRC expressing wild-type KRAS. In 2017, the US FDA expanded the indication to include Vectibix for treating mCRC expressing wild-type RAS (both KRAS and NRAS). The results of both the phase 3 PRIME and ASPECCT trials revealed that Vectibix medication significantly enhanced survival in patients with advanced CRC (Douillard et al., 2010).

From two decades of discussion and clinical research, targeting the EGFR family and its intracellular signaling pathways remains the targeted molecular therapy for mCRC with highest importance. The medicinal application of cetuximab and panitumumab, two anti-EGFR monoclonal antibodies, is restricted to a subset of mCRC patients. Nonetheless, this choice was made due to the absence of prognostic molecular biomarkers of response (i.e., RAS activation or BRAF-activating mutations) rather than the presence of positive predictive biomarkers of antitumor activity and efficacy. Despite the fact that chemotherapy coupled with anti-EGFR monoclonal antibodies has great efficacy in mCRC patients with wild-type left-hemi-RAS/BRAF tumors, with a primary objective response reported in nearly two-thirds of patients and a median PFS of 11 months, all patients developed disease progression. In about one-third of the patients, this was due to emerging RAS mutations and cloning of cancer cells with BRAF mutations or EGFR ectodomain mutations as an escape mechanism from EGFR inhibition (Strickler et al., 2018). Indeed, therapy with anti-EGFR drugs removes RAS/BRAF-sensitive clones of the wild type (RAS/BRAF-wt), and the obtained RAS mutation-resistant clones become the majority population of cancer cells. Consequently, second-line therapy, besides adding an anti-angiogenic agent, usually includes a change in the chemotherapy regimen. However, a growing number of clinical studies suggest (Misale et al., 2014; Parseghian et al., 2019) that anti-EGFR drug retreatment may play a role in the sequential therapy of patients with wild-type RAS/BRAF mCRC. Indeed, the gradual decay of acquired resistant RAS mutant clones (half-life of nearly 4 months) during second-line, EGFR inhibitor-free therapy and the potential proliferation of wild-type RAS/BRAF clones to increase sensitivity to anti-EGFR medications has resulted in the suggestion of re-challenge anti-EGFR therapy. This potential re-introduction of anti-EGFR treatment may be used as a backline medication for patients who are sensitive to first-line anti-EGFR therapy and not medicated with anti-EGFR monoclonal antibodies in second- and third-line therapies.

## 4 Anti-BRAF, MEK-targeted therapy: vemurafenib, dabrafenib, encorafenib, trametinib, and binimetinib

In CRC, the occurrence of BRAF mutation ranges from 10% to 20%. Out of these mutations, around 90% are BRAF V600E locus mutations, accounting for 7%–15%, while non-V600E mutations constitute about 2%. In CRC cases exhibiting high microsatellite instability (MSI-H) or deficiency in mismatch repair protein (dMMR) expression, BRAF V600E mutations often coexist with MLH1 expression deficiency or hypermethylation in the MLH1 gene promoter region. International reports indicate a concurrent rate of about 30%–75% (Jones et al., 2017). In CRC with MSI-H or dMMR, BRAF V600E mutations are often accompanied by MLH1 expression deficiency or MLH1 gene promoter region hypermethylation, with a concomitant rate of about 30%–75% reported abroad (Ward et al., 2013). BRAF and RAS mutations are usually mutually exclusive and linked to females, usually right hemizygous, late-staged, with mucinous histological manifestations, defective mismatch repair, and malignantly formed serrated adenoma. CRC patients with BRAF V600E mutation have a median OS of nearly 11 months, are chemotherapy insensitive, have an extremely poor prognosis, and are poorly treated with standard chemotherapy (Cremolini et al., 2015). BRAF mutations induce self-activation of downstream genes, which cause tumor cell growth and accelerated proliferation.

BRAF-mutated mCRC is an extremely complex subgroup of CRC. In the last decade, certain breakthroughs have been achieved in targeting the BRAF-mutated CRC subgroup, but the optimal recommended strategy for treating BRAF-mutated mCRC patients remains to be determined. Options include single-agent, two-agent, and three-agent chemotherapy, anti-EGFR targeted therapy, BRAF inhibitors, and MEK inhibitors. Besides there are combinations of target and chemotherapy and combinations between two or more targets. However, using chemotherapy with anti-angiogenic drugs in the first line and BRAF inhibitors plus EGFR inhibitors after disease progression is still the approved guideline of care for patients with appropriate physical status.

Vemurafenib, dabrafenib, and trametinib are selective BRAF/MEK suppressors that were created and tested in clinical trials (Grothey et al., 2021). Anti-BRAF monotherapy is poorly effective. Based on the BEACON CRC research, encorafenib combined with cetuximab protocol significantly enhances OS when compared to the control group, with a median OS of 9.3 months when compared with 5.9 months of the control group. When encorafenib and cetuximab were combined with binimetinib, the efficacy was similar; both protocols significantly increased outcomes and quality of life when compared with controls in mCRC patients with BRAF V600E mutations in whom disease progressed on first- or second-line therapy (Kopetz et al., 2015). After progression on first-line therapy for mCRC with BRAF V600E mutations, the NCCN recommendation for subsequent systemic therapy is encorafenib in combination with an EGFR inhibitor such as cetuximab or panitumumab. In the newest NCCN guidelines, the three-drug strategy of dabrafenib and trametinib, encorafenib and binimetinib, and cetuximab and panitumumab for BRAF V600E–mutated mCRC was rejected. BEACON CRC research findings support NCCN recommendations (Corcoran et al., 2018; Kopetz et al., 2019).

Immunotherapy demonstrates potential benefits for patients with BRAF-mutated, microsatellite stable (MSS)–type metastatic CRC (mCRC). Preliminary studies suggest a potential synergy when BRAF inhibitors are combined with PD-1/PD-L1 immune checkpoint inhibitors. One investigation indicated that the pairing of BRAF inhibitors with EGFR monoclonal antibodies encouraged a temporary MSI-H phenotype in MSS-type bowel cancer, hinting at potentially improved survival outcomes for patients harboring BRAF mutations. A recent ASCO meeting unveiled a phase I/II study that combined cetuximab and nivolumab in MSS-type, BRAF V600E–mutated mCRC (Morris et al., 2022). In total, 26 patients were included, and 24 evaluable patients achieved an overall remission rate of 50%, DCR of 96%, median PFS of 7.4 months, and median OS of 15.1 months. This is the best outcome achieved so far in second-line treatment and beyond for patients with BRAF mutations, and the randomized phase II SWOG 2107 research is also ongoing. In addition, because BRAF mutations often lead to sustained activation of the RAS/RAF/MEK/ERK pathway, co-inhibition of BRAF and MEK contributes to pathway suppression, and combined immunotherapy on this basis may be more efficacious. Dabrafenib and trametinib with PD-1 inhibitors are being researched together to provide further clinical guidance.

## 5 Anti-HER2 therapy: trastuzumab, pertuzumab, lapatinib, and tucatinib

Overexpression of HER2 accounts for 2%–3% of all CRC (Yaeger et al., 2018), commonly found in patients having left-hemicolectomy colon cancer, RAS-wt. It is currently believed that HER2 overexpression status may be a negative indicator of anti-EGFR monoclonal antibody effectiveness (Sartore-Bianchi et al., 2019). However, a proportion of HER2-amplified mCRC patients may still benefit from EGFR monoclonal therapy, and it is also a biomarker to guide anti-HER2-targeted medication in progressed CRC. Moreover, HER2 immunohistochemical testing is recommended in all mCRC. The determining criteria for HER2 overexpression in CRC mainly refer to the HERACLES study (Sartore-Bianchi et al., 2016), which requires tumors with a 3+ HER2 score in over 50% of cells by immunohistochemistry or with a 2+ HER2 score and 50% of tumor cells to be FISH positive. With the development of NGS, some studies have used NGS detection of increased HER2 gene copy number as a criterion for HER2 overexpression interpretation as well. For patients with advanced CRC with HER2 overexpression, anti-HER2 therapy is recommended as a second-line treatment.

A number of clinical studies resulting from the identification of HER2 gene amplification in a subset of patients with wild-type RAS/BRAF mCRC (Bertotti et al., 2011) have been conducted that evaluated different anti-HER2 treatment strategies. Treatment with the humanized anti-HER2 monoclonal antibody trastuzumab plus the anti-HER2 tyrosine kinase inhibitor lapatinib is the recommended and effective treatment for patients with HER2-amplified chemo-refractory mCRC. Additional potential strategies include combining trastuzumab and the humanized anti-HER2 monoclonal antibody pertuzumab, as well as the use of heavyweight antibody–drug conjugates (ADCs) such as Enhertu (trastuzumab deruxtecan) (Sartore-Bianchi et al., 2020; Siena et al., 2021). In addition, antitumor activities were observed with trastuzumab in combination with the selective anti-HER2 tyrosine kinase inhibitor tucatinib (Gao et al., 2022). The 2022 ASCO meeting reported that MOUNTAINEER was an open-label, multicenter phase 2 clinical research in the United States and Europe, enrolling 117 HER2-positive patients not eligible for surgery or mCRC patients who had experienced standard treatment before but not received anti-HER2 therapy. In the trial, patients were medicated with Tukysa plus trastuzumab or Tukysa monotherapy. The overall remission rate for CRC patients receiving tucatinib plus Herceptin was 38.1%, with a median duration of remission of 12.4 months, median PFS of 8.2 months, and median OS of 24.1 months. The US FDA has accepted its new drug tucatinib/Tukysa plus trastuzumab for priority approval eligibility in the treatment of patients with HER2-positive CRC who have undergone at least one past therapy for disease that cannot be resected or developed metastasis. Tukysa is an oral HER2 tyrosine kinase inhibitor that expresses HER2 in tumor cells showing antitumor activity. *In vivo*, Tukysa inhibits the growth of tumors with HER2 expression. Trastuzumab plus Tukysa have demonstrated better antitumor activity than either agent alone. As anti-HER2 therapy effectiveness has been established in treating chemotherapy-refractory patients, clinical studies of this class of medications are now evaluating their potential involvement in the early phases, such as in first-line treatment. Therefore, a global phase 3 randomized clinical trial, MOUNTAINEER-03, has been started with the aim to determine the effectiveness of Tukysa plus trastuzumab, standard chemotherapy in the presence or absence of cetuximab and bevacizumab, as the first-line medication for HER2-positive mCRC (42-44).

## 6 Targeted KRAS mutation therapy

The RAS family consists of three variant genes encoding four proteins: HRAS, NRAS, KRAS4A, and KRAS4B (the latter two being isoforms generated by distinct splicing), as KRAS4B is the major splice variant, also referred to as KRAS (Simanshu et al., 2017), and the presence of KRAS-activating mutations is identified in mCRC in anti-EGFR therapy as the first predictive negative biomarker.

Studies have proven that mutation of KRAS exon 2 activates MAPK signaling that bypasses the upstream blockade of EGFR by the therapeutic monoclonal antibodies cetuximab or panitumumab (Karapetis et al., 2008). Recent research has demonstrated that, in addition to KRAS exon 2, other KRAS and NRAS mutations are resistant to anti-EGFR therapy (Van Cutsem et al., 2015). Over 40% of mCRCs have KRAS mutations, specifically common in exon 2 and codon 12 (approximately 80% of all KRAS mutations) and codon 13 and in exons 3 (codons 59 and 61) and 4 (codons 117 and 146). NRAS mutations are rarer (5%–10% of mCRC) and occur mainly in exons 3 (codon 61) and 2 (codons 12 and 13) (Nassar et al., 2021). RAS mutations are usually linked to poor outcome and drug resistance.

KRAS G12C mutation has been on the radar of cancer researchers since 40 years ago, but drug development for this target has been slow that it was once considered a “non-druggable target.” However, since 2013, several selective and irreversible inhibitors targeting KRAS G12C mutation, which occurs in about 3%–4% of mCRC patients, have been under development (Jones et al., 2017). Sotorasib and adagrasib were first accepted by the US FDA for treating KRAS G12C–mutated advanced lung cancer and were subsequently studied in refractory advanced studies in colon cancer (Hong et al., 2020; Kim et al., 2020; Fakih et al., 2022) showed that sotorasib and adagrasib had single-agent efficiencies of 10%–22%, achieving a breakthrough in targeted therapy for RAS-mutant colon cancer. The 2022 ESMO Congress KRYSTAL-1 has reported that the trial evaluated the effectiveness and safety of the KRAS G12C inhibitor adagrasib, and this congress has presented results of its CRC single-arm analysis of adagrasib (MRTX849) in combination with or without cetuximab in patients with KRAS G12C–mutated progressed CRC, with 44 patients receiving adagrasib monotherapy and 32 patients receiving a combination of adagrasib and cetuximab in the single-agent group with an overall remission rate (ORR) of 19%, a disease control rate (DCR) of 86%, and a median duration of remission (DOR) of 4.3 months (95% CI, 2.3–8.3). The median PFS of the patients was 5.6 months (95% CI, 4.1–8.3) while the median OS was 19.8 months (95% CI, 12.5–23.0). Patients in the combination therapy group had an ORR of 46%, DCR of 100%, median DOR of 7.6 months (95% CI, 5.7–NE), median PFS of 6.9 months (95% CI, 5.4–8.1), and median OS of 13.4 months (95% CI, 9.5–20.1). Adagrasib alone and in combination with cetuximab showed good clinical activity in KRAS G12C–mutated advanced CRC patients with a manageable safety profile. The investigators noted that the combination caused higher remission rates and longer PFS, and a follow-up phase III study of this combination strategy S.J. et al. (2022), Professor James K. Chen’s team, Li Jiong and Teng Xiu’s team, and Yu Jun’s team (KRYSTAL-10) is underway.

The investigators also mentioned that two phase III trials are currently evaluating the efficacy of these combinations in patients with KRAS G12C–mutated CRC. Adagrasib in combination with cetuximab versus chemotherapy is being investigated in KRYSTAL-10 as second-line therapy in mCRC (NCT04793958), and CodeBreak 300 (NCT05198934) is evaluating sotorasib in combination with panitumumab with investigator-selected therapies (trifluridine and tipiracil, or regorafenib) in previously treated metastatic CRC, which may include third-line therapy. Although this is a novel approach to treat KRAS G12C–mutated advanced CRC, only this small subset of KRAS-mutated patients will benefit, as most other patients with KRAS-mutated types remain untargeted, and further work is required to understand the biology of KRAS-mutated CRC, to directly target other KRAS mutations with new selective agents and combinations, and metabolic pathway inhibitors, to indirectly target MAPK signaling (Klempner et al., 2022; Kuboki et al., 2022).

## 7 NTRK gene fusion targeted therapy

NTRK fusion (Pietrantonio et al., 2017) is a very rare mutation in mCRC, with an incidence of 0.20%–1%, but has a higher incidence of about 5% in MSI-H CRC, indicating the possibility of testing for NTRK fusions in patients with dMMR/MSI-H tumors. In recent years, two “cancer-free” therapies (entrectinib and larotrectinib) have been authorized by the US FDA and European Medicines Agency (EMA) that are based on the presence of a specific mutation in the NTRK fusion (independent of tumor type). The 2022 ESMO Congress reported (Doebele et al., 2020) that among 34 patients enrolled for larotrectinib treatment, which included 19 cases of CRC, pancreatic, bile duct, appendix, gastric, liver, esophageal, and other gastrointestinal tract tumors, the ORR was 33%, with a complete remission (CR) rate of 3% and partial remission (PR) rate of 30%, with 45% of patients having stable disease. It is notable that among the 19 CRC patients, the ORR was 47% and CR rate was 5%, which means that about 50% of the CRC patients had a significant reduction in or even disappearance of lesions. An additional 42% of the patients had stable disease, with a disease control rate of 89%.

At the 2022 ESMO World Congress on Gastrointestinal Cancers (WCGIC 2022) (Garrido-Laguna et al., 2022), the Huntsman Cancer Institute at the University of Utah in Salt Lake City revealed the findings of a recent comprehensive analysis exploring the use of entrectinib in patients with NTRK fusion-positive gastrointestinal tract tumors and found that entrectinib had durable efficacy in these patients. Of 16 objectives with different types of gastrointestinal tract tumors, 10 cases had CRC, and the remission rate was 40% for the entire cohort of patients with gastrointestinal tract tumors and approximately 20% for patients with CRC, with a duration of remission of nearly 20 months and a PFS of nearly 7 months. The OS of patients in this cohort was 4 months longer than was previously reported.

## 8 Targeted RET inhibitors

RET, an oncogene, encodes a transmembrane receptor equipped with a tyrosine kinase structural domain. Mutations or fusions involving RET can incite downstream signaling pathways such as RAS/MAPK, PI3K/AKT, or JNK, thereby promoting cell survival and tumor growth (Jhiang, 2000). In a study that sequenced 39 different histological types and a total of 4,871 tumor tissue samples, only 1.8% of solid tumors had RET gene variants, of which the gene fusion types accounted for 30.7% (Kato et al., 2017). However, the incidence of RET fusions in patients with CRC was less than 1% (Gourd, 2018). Although RET fusions were rare variants in CRC, one study found that more than two-thirds of the patients with right hemizygous colon cancer, RAS/BRAF wt, and MSI-H in CRC carried RET gene fusions. Furthermore, this study found that RET fusion-positive patients had a poorer prognosis and OS than did RET fusion-negative patients (median OS 14.0 months vs. 38.0 months, HR: 4.59; *p* < 0.001) (Pietrantonio et al., 2018). In mCRC, RET fusions can be potential therapeutic targets and prognostic markers.

The 2022 ASCO guidelines for CRC both added the first treatment option for patients with positive RET gene fusions. Selpercatinib is a highly selective RET tyrosine kinase inhibitor. On 21 September 2022, according to the research findings of LIBRETTO-001 (NCT03157128) (Subbiah et al., 2022), the FDA accelerated the approval for the use of selpercatinib in adult patients with locally advanced or metastatic solid tumors with disease progression or fusion of RET genes without other satisfactory alternative treatment options after previous systematic treatment. The LIBRETTO-001 study was a global multicenter, phase 1/2, open-label, basket study that investigated the efficacy and safety of selpercatinib in patients with RET fusion-positive advanced solid tumors (excluding lung or thyroid cancer). A total of 45 patients (10 with colon cancer) were enrolled, and the efficacy analysis showed an ORR of 43.9%, which included 20.0% in patients with colon cancer. Regarding safety, 18 patients (40%) experienced serious on-treatment emergent adverse events (TEAEs), with the most common grade ≥3 TEAEs of hypertension and abnormal liver functioning (elevated ALT and AST). Owing to the small sample size of this research, with limited clinical data available to evaluate patients with colon cancer, and the fact that selpercatinib was only owing to the limited sample with 114, the validation of the results of this study in confirmatory clinical trials with larger sample sizes is still required. Therefore, selpercatinib was not included in the treatment pathway for mCRC in this update of the NCCN Colon and Rectal Cancer Guidelines but only added as a footnote.

## 9 Other new targets

### 9.1 ALDH1B1, acetaldehyde dehydrogenase

Ethanol dehydrogenase (ADH) in the liver catalyzes the conversion of ethanol (alcohol) to acetaldehyde, and acetaldehyde dehydrogenase (ALDH) is responsible for further catalyzing the resulting acetaldehyde to harmless acetic acid. In humans, the ALDH family comprises several isozymes. Prior investigations (Feng et al., 2022) have detected the presence of cancer cells with high levels of ALDH activity in a variety of cancers, and such cancer stem cells are more tumorigenic, chemoresistant, and metastatic. For example, high levels of ALDH1A3 are present in breast, glioma, melanoma, and non–small-cell lung cancer stem cells, while ALDH1B1 are highly expressed in cancer stem cells of CRC and pancreatic ductal adenocarcinoma (PDAC).

A study led by Professor James K. Chen's team at Stanford University resulted in the development of IGUANA-1, a selective inhibitor of ALDH1B1. IGUANA-1 curbs the proliferation of CRC cells and organoids, underscoring the significant role that ALDH1B1 plays in CRC. This finding indicates that inhibitors of ALDH1B1 could potentially serve as a therapeutic strategy for CRC, introducing a new direction in the development of targeted drugs against ALDH1B1.

### 9.2 Interleukin 37

Interleukin 37 (IL-37) was discovered to be an important natural and acquired immunity suppressor. In CRC, the molecular mechanism and role of IL-37 have remained obscure. In 2022, Li Jiong and Teng Xiu's team at Sichuan University (Wang et al., 2022) observed that IL-37 transgenic (IL-37tg) mice were highly sensitive to CAC and had significantly elevated colonic tumor burden. However, in intestinal mutagenesis, CRC cell aggressiveness did not require IL-37. Importantly, IL-37 blocked cytotoxic T-cell-mediated immunity in CAC and B16-OVA models. Finally, this study observed significantly elevated IL-37 levels in CRC patients that were positively linked to serum CRC biomarker CEA levels but negatively linked to CD8^+^ T-cell infiltration in patients. In conclusion, the results of this study have highlighted the function of IL-37 in harnessing antitumor immunity through the inactivation of cytotoxic T cells and established a newly defined suppressor IL-37/SIGIRR as a treatment option for CRC in the cancer immune cycle.

### 9.3 Squalene epoxidase

Huang et al. (2020) have shown that excessive cholesterol intake increases the risk of CRC and that squalene epoxidase (SQLE) is the rate-limiting enzyme in cholesterol biosynthesis. Yu Jun's team at the Chinese University of Hong Kong published a study in the journal *Gut* and found that SQLE mRNA and protein expression were upregulated and predicted poor survival in CRC patients (Li et al., 2022). SQLE enhanced CRC cell growth by triggering cell cycle progression and inhibiting apoptosis. In the azomethane-induced CRC model, colon-specific SQLE transgenic (tg) mice (Sqle tg) had elevated tumorigenesis, while Sqle KO mice had decreased tumor numbers in the colon when compared with wild-type mice. Integrated macrogenomic and metabolomic analyses have revealed a dysregulated intestinal ecology in Sqle tg mice enriched in pathogenic bacteria, which was associated with increased secondary bile acids. In accordance with the harmful consequences of secondary bile acids, Sqle tg mice have impaired intestinal barrier functioning and decreased tight junction protein Jam-c and occludin. Finally, this study revealed that terbinafine, an SQLE inhibitor, could be repurposed for CRC by synergistically inhibiting CRC growth with oxaliplatin and 5-fluorouracil. To conclude, this research has suggested that SQLE mediates tumorigenesis through cell-intrinsic effects and regulation of the metabolite axis of the gut microbiota. The studies have suggested that SQLE is a possible treatment option and prognostic marker in CRC.

Additionally, immunotherapy is an encouraging route to explore in CRC treatment. Research has demonstrated that immunotherapy is efficacious and safe for mCRC patients who are dMMR/MSI-H. Nonetheless, the vast majority of mCRC patients (approximately 85%–90%) present with MSS and normal mismatch repair (pMMR). Despite the existence of limited studies with small cohorts, there is optimism that immunotherapy can make significant strides in the area of MSS/pMMR in the coming years. When compared with the wide application of PD-1/PD-L1 inhibitors in tumor therapy, CTLA-4 monoclonal antibodies have been used as a single drug and in combination with other ICIs. Currently, only ipilimumab is approved by the US FDA for the clinical treatment of tumors, and it is the first drug that was proven to prolong OS in patients with advanced melanoma (McDermott et al., 2013). In a clinical trial (CheckMate 142) (Overman et al., 2017), 119 patients with metastatic mCRC—who had been previously treated with fluorouracil and oxaliplatin or irinotecan—and MSI-H/dMMR received four cycles of “O + Y” combination therapy, followed by 3 mg/kg sequential nivolumab every 2 weeks until progression or death. The BRAF and KRAS mutation rates of the enrolled patients were 24% and 37%, respectively, and the ORR was 49%, of which the rate of CR and PR was 4% and 45%, respectively; the “O + Y” combination therapy was approved by the US FDA. A clinical study (Derakhshani et al., 2021) indicated an overexpression of CTLA-4 in the CRC tissues when compared to the adjacent non-tumoral tissues and that SW480 cells substantially overexpress CTLA-4 when compared to HCT 116 and HT-29 cells. In addition, capecitabine remarkably downregulates the expression of CTLA-4. Collectively, capecitabine can inhibit the expression of CTLA-4 in CRC cells and might bridge immunotherapy approaches with chemotherapy. Thus, we know the transformative potential of CTLA-4 inhibitors in reshaping the therapeutic landscape of mCRC. However, at present, only ipilimumab and tremelimumab have been approved as CTLA-4 inhibitors. Ipilimumab is only approved for monotherapy in specific cases of melanoma, and the rest are combination therapies, which are still characterized by a small number of drug classes, narrow range of indications, and lack of optimal dosage and treatment strategies. In addition, there is a requirement to be deeply aware of the complexity, uncertainty, and risk of immunotherapy. Attention should be paid to the occurrence of AEs in anti-CTLA-4 monotherapy and “O + Y” combination therapy. Early clinical trials mostly used high-frequency, high-dose ipilimumab monotherapy, which showed that the incidence of AEs was as high as 53% and the discontinuation rate was 28%, which was higher than that of the chemotherapy group, and only 46% of the patients in the ipilimumab group completed the treatment (Govindan et al., 2017). CTLA-4 inhibitors represented by ipilimumab have been on the market for many years but failed to have a major breakthrough in a variety of solid tumors, probably because the mechanism of action of CTLA-4 is still unclear, and with the emergence of PD-1/CTLA-4 combination, therapeutic agents could solve the aforementioned problems. In conclusion, due to the complexity, uncertainty, and certain risk of immunotherapy, more sufficient evidence-based medical research is still required to screen the advantageous population of colorectal cancer immunotherapy through accurate biomarkers and predict the efficacy and risk of immunotherapy accurately. The combined use of CTLA-4 inhibitors in different stages and treatment should be carried out prudently under the guidance of evidence-based medicine and relevant domestic and international guidelines.

## 10 Conclusion

With in-depth research on the pathophysiology of CRC and its treatment, the discovery of novel targets can help corresponding drugs that are designed for specific targets in patients with especially CRC who are prolonged under the guidance of individualized precision treatment. It improves the overall survival and quality of life. However, there are still many problems in targeted therapy. The focus of future research is on the efficacy of targeted drugs in evaluation, besides existing clinical efficacy assessment criteria for solid tumors (response evaluation criteria in solid tumor—RECIST), how to combine other imaging signs before and after treatment, marker changes, establishing a set of drugs that are more suitable for targeted therapy, and evaluation criteria.. How to predict the targeting efficacy of drugs before treatment and to find effective biomarkers are also the main challenges and goals of the front. In addition, anti-angiogenesis is better controlled adverse effects associated with targeted drugs are also of concern. Currently, there are more and more clinical trials that explore the potential for targeted drugs with different mechanisms of action suitable for application areas, such as targeted therapy with chemotherapy and different mechanisms. With the progression of the combination of therapy, including chemotherapy, targeted terapy and immunue therapy for cancers. Believing in targeted cure therapeutic drugs will have a more essential part in comprehensive medication of CRC in the future.
